# Microinvasive mitral valve surgery: Current status and status quo

**DOI:** 10.3389/fcvm.2023.1094969

**Published:** 2023-04-26

**Authors:** Anton Tomšič, Robert J. M. Klautz, Michael A. Borger, Meindert Palmen

**Affiliations:** ^1^ Department of Cardiothoracic Surgery, Leiden University Medical Centre, Leiden, Netherlands; ^2^ Leipzig Heart Center, University Clinic for Cardiac Surgery, Leipzig, Germany

**Keywords:** mitral valve, mitral valve repair, microinvasive mitral valve surgery, mitral valve prolapse, cardiac surgery

## Abstract

Surgical mitral valve repair, performed either through median sternotomy or minimal invasive approach, presents the gold standard treatment for degenerative mitral valve disease. In dedicated centres, high repair and low complication rates have been established with excellent valve repair durability. Recently, new techniques have been introduced, that allow mitral valve repair to be performed through small surgical incisions and while avoiding cardio-pulmonary bypass. These new techniques, however, conceptually differ significantly when compared to surgical repair and it remains questionable whether they are capable of reproducing the results of surgical treatment.

## Introduction

Growing interest in reduction of surgical trauma and improved cosmetic results has resulted in the development of minimal invasive surgical techniques. In case of mitral valve surgery, thoracoscopic and, to a lesser extent, robot-assisted surgical techniques are nowadays used and are, in selected patients, capable of reproducing the techniques and results of conventional mitral valve surgery (1, 2). Regardless of the surgical approach, mitral valve surgery is a safe operation with excellent repair durability when performed in experienced centres (3). Lately, even less invasive surgical [so-called “micro-invasive” (4)] and transcatheter techniques have emerged as an alternative to the established surgical techniques. Beating heart implantation of neochords is one the most commonly used such techniques. Several devices are nowadays commercially available, but all follow the basic principle of resolving leaflet prolapse by chordal replacement therapy. The surgical procedure is performed through a left-sided mini-thoracotomy incision on beating heart and with transoesophageal echocardiographic guidance. Furthermore, a transseptal transcatheter-based approach to neochord insertion is being developed. Avoiding the use of cardiopulmonary bypass and hereto related complications while minimizing surgical trauma seems a promising goal for these procedures. However, surgical and beating heart mitral valve repair are conceptually two very different treatment options. Limitations exist and it remains questionable if the results of surgical repair can be replicated in this setting.

## The basic principles of surgical mitral valve repair

The goal of reconstructive mitral valve therapy is unrelated to treatment modality (surgery or transcatheter) or type of surgical access. Resolution of mitral insufficiency without any residual regurgitation remains the primary goal of therapy. Moreover, a stable repair without recurrence of mitral regurgitation will lead to optimal clinical results. In technical terms, the strategy of mitral valve repair remains based on the pioneering principles of Carpentier: (I) preserve or restore normal leaflet motion, (II) create a large surface area of leaflet coaptation, and (III) remodel and stabilize the mitral valve annulus (5). Conceptually, surgical repair treats the mitral valve as a comprehensive functional complex consisting of the leaflets, subvalvular apparatus, annulus and the adjacent left ventricle and left atrium. Abnormalities or dysfunction of one or more of these parts will affect other components, and therefore addressing all different parts of the mitral apparatus following Carpentier's rules is crucial for good surgical valve repair durability. On the other hand, beating heart mitral valve repair indirectly implies that the mitral valve complex consists of independent units that can be addressed individually. Focusing on a single leaflet or, more common, prolapsing scallop and resolving excessive leaflet motion by implanting artificial neochords is a fundamentally different approach when compared to surgical valve repair.

## The importance of the mitral valve annulus

**Figure 1:**
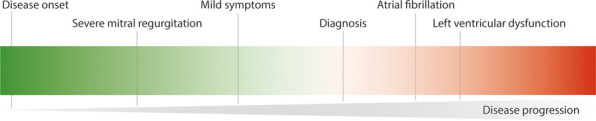
Degenerative mitral valve disease is a progressive disease and the majority of patients are diagnosed once symptoms or other complications arise.

The mitral annulus plays an important role in the pathogenesis of degenerative mitral valve disease. With time, annular dilation and flattening occurs, with loss of sphincter-like function of the annulus. Moreover, progressive leaflet growth and myxomatous degeneration is characteristic for degenerative valve disease (6). This is important to take into account when assessing the degree of annular dilation in these patients. Arbitrarily, this is present when the ratio between the anterior-posterior annular diameter and anterior leaflet length is >1.3 or when the diameter is >35 mm (7). In the presence of enlarged mitral valve leaflets, a favorable ratio between the anterior-posterior annular diameter and anterior leaflet length will not reliably exclude underlying mitral annular geometric and functional remodeling. From a clinical perspective, it is important to realize that these changes will precede the diagnosis of severe mitral valve disease (Figure 1).

Sufficient length of leaflet coaptation is crucial to ensure valve repair durability (8). With sufficient length of coaptation, the forces of valve closure will be transduced to the coaptating area of the mitral valve leaflets. This will ensure that the stress experienced by the mitral valve apparatus at the time of valve closure will not be transduced to the primary chordae, that are primarily responsible for appropriate leaflet apposition in the early phase of valve closure. In theory, failure to perform annular remodeling and stabilization, with ensuing insufficient length of leaflet coaptation, will thus lead to suboptimal valve closure properties. This predisposes to rupture of the primary chordae and recurrent leaflet prolapse, and underlines the importance of appropriate annuloplasty ring or band sizing. Of note, complete annular reverse remodeling with recovery of annular sphincter function is unlikely to occur after sole resolution of mitral regurgitation. This is supported by the limited amount of annular size reduction after transcatheter edge-to-edge repair for degenerative disease (9).

The debate on the need for surgical annuloplasty remains ongoing (10). While several criteria, based on geometric valve properties, have been proposed to identify patients without significant annular dysfunction, little supporting evidence on the durability of valve repair without annular remodeling and stabilization is available. Moreover, the mitral valve annulus is a dynamic structure with three-dimensional properties, including the annular saddle shape, that have and important effect on leaflet stress during valve closure. Proper identification of patients with intact annular geometry and dynamics despite severe regurgitation might be related to more detailed assessment of annular movement prior to surgery. The number of patients in whom, even theoretically, valve repair without annuloplasty is justified is most likely limited.

## Preoperative planning

Detailed transthoracic and transesophageal echocardiography is crucial to identify patients suitable to undergo this procedure. General contraindications, either patient- (e.g., the need for concomitant procedures) or valve-related (e.g., mitral annular or leaflet calcification), are easily identifiable. On the other hand, identification of patients in whom a durable repair is feasible remains elusive. Several indexes, including the tissue-to-gap ratio and leaflet-to-annulus index, to reliably predict sufficient height of leaflet coaptation after the procedure, have been proposed (11, 12). While these might help improve early outcomes, the effect of their implementation on late repair durability remains to be proven.

## Results of beating heart mitral valve repair

Experience with beating heart mitral valve repair with chordal replacement remains limited at this time. The available data are based on the use of the Neochord DS 1000 (Neochord Inc., St. Louis Park, MN, USA) and Harpoon TDS-5 devices (Edwards Lifesciences, Irvine, CA, USA) devices (13). Moreover, the first comparative studies comparing the technique with conventional surgical repair have only recently been published (14). No data from randomized controlled trials are available to date. An overview of the largest published series is presented in Supplementary Table S1.

Report of an early European experience, including 213 patients who underwent mitral valve repair with the NeoChord device, demonstrated reduction of regurgitation severity to less than moderate at the time of the procedure in 87% of patients (11). At 30 days post-intervention, however, only 77% of patients were free from recurrent mitral regurgitation. One of the most important observations was the proposed patient stratification based on degenerative disease severity. “Type A” patients with isolated central posterior leaflet prolapse/flail demonstrated best results, but it should be noted that at 30 days after the intervention, recurrent regurgitation was already seen in 10% of patients from this group as well. At 1 year after surgery, recurrent regurgitation was seen in 15% of patients with “Type A” anatomy, excluding the patients who underwent reintervention during this time period. The results were poorest in patients with “Type C” anatomy, referring to patients with anterior, bileaflet or paracommissural disease with/without leaflet and/or annular calcifications. At 1 year after the intervention, recurrent mitral regurgitation was present in 56% of patients, again excluding the patients who underwent reintervention during this time period.

A recent report on the results of beating heart mitral valve repair with the HARPOON System, including 65 patients with isolated posterior leaflet prolapse, demonstrated technical success in 62 patients (95%) (12). 2 patients required intraoperative conversion to conventional mitral valve repair. At discharge, moderate or severe residual mitral regurgitation was present in 5% of patients. However, the frequency of recurrent regurgitation rose to 15% and 25% at 30 days and 1 year after surgery, respectively. Of note, these rates were exclusive of patients who underwent mitral valve reintervention. The rate of reintervention was 2% and 13% at 30 days and 1 year after surgery, respectively.

In a recent single center analysis of 100 patients treated with the NeoChord procedure, the results of beating heart mitral valve repair of 81 patients with favorable anatomy were compared to the remaining 19 patients with unfavorable anatomy (15). For patients with favorable anatomy, the incidence of recurrent severe mitral regurgitation was 6.2%, 7.5% and 14.7% at 1, 3 and 5 years, respectively. No data on freedom from moderate mitral regurgitation were reported.

A recent retrospective study compared the results of isolated conventional mitral valve surgery to beating heart mitral valve repair (14). Propensity score matching was used to balance for differences in baseline characteristics. At discharge, recurrent mitral regurgitation was more frequent in the beating heart mitral valve repair group (9.1% vs. 1.1%). With a follow-up duration extending up to 5 years after the intervention, freedom from mitral regurgitation was worse for the beating heart mitral valve repair group (57.6%, 95% confidence interval 43.0%–77.1% vs. 84.6%, 95% confidence interval 75.6%–94.6%).

Lastly, the mechanism of repair failure after beating heart mitral valve repair differ significantly when compared to surgical valve repair. In particular, high incidence of early disease progression, relative elongation of the implanted neochords due to left ventricular reverse remodeling and posterior leaflet curling are frequently reported as the mechanism of repair failure (15, 16).

## Discussion

The contemporary results of surgical mitral valve repair for degenerative disease are excellent with valve repair rates approaching 100%, early mortality rates below 1%, and low perioperative morbidity in experienced centers (3, 17, 18). Moreover, excellent valve repair durability has been achieved with low reintervention and recurrent mitral valve regurgitation rates (17–19). In terms of clinical outcomes, mitral valve repair is capable of eliminating symptoms related to valve dysfunction and restore normal quality of life and lifespan, provided that surgical intervention is performed in the earlier phases of the disease (18). Surgical mitral valve repair has set a very high standard and reproduction, let al.one improvement, of these results will be hard to achieve.

The excellent results of surgical mitral valve repair result from the concept of the mitral valve apparatus as a comprehensive structure. While surgical techniques have evolved over time, the basic principles of valve repair remain to date unchanged. Surgical repair offers the advantage of direct valve exposure and is able to adapt to unexpected findings and various valve phenotypes. This include abnormalities of the sub-valvular apparatus, potentially hampering normal leaflet motion, that can effectively be addressed at the time of surgical correction. On the other hand, beating heart mitral valve repair with chordal replacement focuses merely on a particular, “diseased” part of the valve. An assumption is made that a durable repair result is feasible in an early phase of the disease. The echocardiographic results published to date bring this assumption into question.

Recurrent mitral regurgitation is not an innocent observation but is associated with adverse cardiac remodeling and a higher risk of death (20, 21). Mild residual regurgitation has been demonstrated as a risk factor for recurrent severe regurgitation after surgical valve repair, showcasing the importance of an optimal initial result to ensure a durable result (20). Moreover, sufficient length of leaflet coaptation is crucial to prevent repair failure (8). Annular remodeling successfully adjusts the mitral valve annulus perimeter to the size of the leaflets and the benefit of annular remodeling and stabilization on valve repair durability is well established (20, 22–24). Recent evidence even suggests that the type of annuloplasty should potentially be adjusted to the location of leaflet prolapse, a showcase of the versatility and potential of improvement of surgical valve repair results (25). The number of comparative studies on the results of valve repair with various annuloplasty devices is limited and warrens further research.

The high incidence of early repair failure after beating heart valve repair with common mechanisms of failure including reverse remodeling of the left ventricle and disease progression, suggest that insufficient leaflet coaptation is achieved with this technique. This leads to unresolved stress exerted by the leaflets, promoting disease progression, or directly to recurrent prolapse, following expected left ventricular reverse remodeling. Pseudo-elongation of artificial neochords has been described for patients following surgical mitral valve repair as well (26). However, such cases are rare as the length of leaflet coaptation achieved with annular remodeling is likely sufficient to prevent the occurrence of this complication. The effect of leaflet coaptation length on surgical repair durability is insufficiently explored. Recent evidence suggest that a longer coaptation length is related to improved echocardiographic outcomes (27). In general, a coaptation length of at least 8 mm should be guaranteed for optimal results.

The net benefit of a treatment presents a balance between its side effects and the clinical improvements that it brings to the patient. Cardiac surgery is and will remain an invasive treatment with increased complications in the early postoperative period when compared to transcatheter therapies. However, a reliable resolution of the underlying problem carries a large upside to the patient that clearly outbalances the early postoperative risks of treatment in the long run, particularly in younger and lower risk patients with a long life expectancy. The reason behind the excellent results of surgical mitral valve repair are high repair rates, reproducibility, and repair durability that all contribute to a better prognosis when compared to valve replacement (28). In case of beating heart mitral valve repair, mitral valve reintervention has been reported in approximately 10% of patients within 1 year and the reported mitral valve replacement rate at reintervention is approximately 50%. Such result not only highlight the frequency of recurrent valve dysfunction but also raise concerns about the feasibility of valve re-repair in case of recurrent regurgitation. The available data do not seem to support the idea that a surgical re-repair is a reliable treatment option in case of failed beating heart mitral valve repair, robbing the patients of the possibility of a durable repair.

In patients after failed surgical mitral valve repair, the beating heart mitral valve repair is an interesting treatment option as annuloplasty band or ring is already in place. Encouraging results in this specific subset of patient have already been reported (29). In particular, good freedom from recurrent mitral regurgitation, with a durable resolution of mitral regurgitation in the majority of patients up to 2 years of surgery, has been reported. Together with high-risk patients who are deemed poor surgical candidates, patients after failed surgical valve repair present a group of patients who are most likely to truly benefit from this technology.

Nationwide studies from the United States demonstrate that the quality of reconstructive mitral valve surgery is good (30). However, with a reported valve repair rate of 80% for degenerative disease, there is room for improvement. The problem of valve repair reproducibility remains an important limitation that would be best addressed with further centralization of care and establishment of dedicated valve centres (31). Innovation and introduction of new surgical and trans-catheter techniques will change the way we treat patients in the future. Beating heart mitral valve repair present an interesting treatment option that will need to evolve in the future to really compete with surgical repair.
