# Supplementary material for: Microinvasive mitral valve surgery: Current status and status quo
Source: Front Cardiovasc Med. 2023 Apr 26;10:1094969. doi: 10.3389/fcvm.2023.1094969 (PMC10169618; doi:10.3389/fcvm.2023.1094969)
Supplement: Supplementary file 1 [file Table1.pdf]

Supplementary table I. An overview of the largest studies on the performance of beating heart mitral valve repair.

Study characteristics			Study population characteristics			Early results				Late results		
	Study design	Number of patients	EuroSCORE II score	STS score	Age	Early mortality	Conversion/early reintervention	Repair rate at early reintervention	Discharge MR grade ≥2+	MR grade ≥2+ at 30 days	MV reintervention at 1 year	Reported repair rate at late reintervention
D'Onofrio et al., 2022 (15)	Retrospective, single-arm	100	1.4% (IQR 0.7-2.3%)	1% (IQR 0.4-1.8%)	66 (IQR 58-76)	2 (2)	3 (3)	N/A	15 (15)	N/A	7 (7)	2/10
D'Onofrio et al., 2022 (14)	Retrospective, propensity score matched	169 vs. 112; 88 matched pairs	0.8% (IQR 0.6-1.5%) vs. 0.7 (IQR 0.6-1.0%)	N/A	63 (IQR 54-72) vs. 63 (IQR 54-72)	0 (0) (PS matched)	1 (1.1)	1/1 (100)	8 (9.1) (PS matched)	N/A	8 (9.1)	5/11
Gammie et al., 2021 (12)	Prospective, single-arm	65	1.2% ± 1.1%	0.6% ± 0.6%	61 ± 12	1 (1.5)	2 (3.1)	2/2 (100)	3 (4.6)	9 (13.8)	8 (12.3)	6/8 (75) (MVR in 1 IE case)
Colli et al., 2018 (11)	Retrospective, single-arm	213	1.8% ± 2.5%	1.5% ± 2.1%	68 (IQR 56-77)	3 (1.9)	4 (1.9)	1/4 (25)	28 (13.1)	48 (22.5)	14 (8.5)	7/14 (50)

Data are presented as N (%), means ± standard deviation or or medians with IQR. Abbreviations: EuroSCORE European System for Cardiac Operative Risk Evaluation; IQR: interquartile range; MR: mitral valve regurgitation; MV: mitral valve; STS: Society of Thoracic Surgeons
